# Elevated Risk of Chronic Respiratory Conditions within 60 Days of COVID-19 Hospitalization in Veterans

**DOI:** 10.3390/healthcare10020300

**Published:** 2022-02-04

**Authors:** Catherine Park, Javad Razjouyan, Nicola A. Hanania, Drew A. Helmer, Aanand D. Naik, Kristine E. Lynch, Christopher I. Amos, Amir Sharafkhaneh

**Affiliations:** 1 VA HSR&D, Center for Innovations in Quality, Effectiveness and Safety, Michael E. DeBakey VA Medical Center, Houston, TX 77030, USA; catherine.park@bcm.edu (C.P.); drew.helmer@va.gov (D.A.H.); aanand.naik@va.gov (A.D.N.); amirs@bcm.edu (A.S.); 2 Big Data Scientist Training Enhancement Program (BD-STEP), VA Office of Research and Development, Washington, DC 20420, USA; 3 Department of Medicine, Baylor College of Medicine, Houston, TX 77030, USA; hanania@bcm.edu (N.A.H.); chris.amos@bcm.edu (C.I.A.); 4 VA Quality Scholars Coordinating Center, IQuESt, Michael E. DeBakey VA Medical Center, Houston, TX 77030, USA; 5 Section of Pulmonary, Critical Care and Sleep Medicine, Baylor College of Medicine, Houston, TX 77030, USA; 6 Department of Management, Policy and Community Health, UT School of Public Health, UTHealth Consortium on Aging, University of Texas Health Science Center, Houston, TX 77030, USA; 7 VA Salt Lake City Health Care System and Division of Epidemiology, University of Utah, Salt Lake City, UT 84132, USA; kristine.lynch@va.gov

**Keywords:** COVID-19, hospitalization, respiratory condition, propensity score matching

## Abstract

SARS-CoV-2 infection prominently affects the respiratory system, and patients hospitalized with COVID-19 are at an increased risk of developing respiratory conditions. We examined the risk of new respiratory conditions of COVID-19 among hospitalized patients in the national Veterans Health Administration between 15 February 2020 and 16 June 2021. The study cohort included all COVID-19-tested, hospitalized individuals who survived the index admission and did not have any previously diagnosed chronic respiratory conditions (asthma, bronchitis, chronic lung disease, chronic obstructive pulmonary disease (COPD), emphysema, or venous thromboembolism) before SARS-CoV-2 testing. Of 373,048 patients hospitalized after SARS-CoV-2 testing, 18,686 positive and 37,372 negative patients met the inclusion/exclusion criteria and were matched by age, sex, and race using propensity score matching. The results showed that the SARS-CoV-2 positive group had a greater risk of developing asthma (adjusted odds ratio (aOR) = 1.37), bronchitis (aOR = 2.81), chronic lung disease (aOR = 2.14), COPD (aOR = 1.56), emphysema (aOR = 1.52), and venous thromboembolism (aOR = 1.92) within 60 days after the index COVID date of testing. These findings could inform that the clinical care team considers a risk of new respiratory conditions and address these conditions in the post-hospitalization management of the patient, which could potentially lead to reduce the risk of complications and optimize recovery.

## 1. Introduction

Coronavirus disease 2019 (COVID-19), caused by severe acute respiratory syndrome coronavirus 2 (SARS-CoV-2), is a highly infectious disease [1,2]. Patients hospitalized with COVID-19 are at high risk of developing acute respiratory conditions [3,4], such as pneumonia, respiratory failure, and acute respiratory distress syndrome (ARDS), which can often lead to death, especially in at-risk and older adults [5]. Observational studies have reported that patients with COVID-19 are at high risk of developing respiratory conditions that can persist after COVID-19 recovery [6,7,8,9,10]. These studies have been limited to small samples, lacked an optimal comparison group, and failed to exclude patients with previous chronic respiratory conditions of patients.

The objective of this retrospective study is to evaluate the risk of developing new respiratory conditions (asthma, bronchitis, chronic lung disease, COPD, emphysema, and venous thromboembolism (VTE)) in associationwith COVID-19 by comparing patients with and without SARS-CoV-2 infection hospitalized in the Veterans Health Administration (VHA).

## 2. Materials and Methods

This is a retrospective study using VHA’s Corporate Data Warehouse (CDW) for all VHA facilities [11] and VHA COVID-19 shared data resources. It contains information on patients tested for SARS-CoV-2 (e.g., timing and nature of test results, medical history, interventions, health conditions, and outcomes). The protocol was approved by the Baylor College of Medicine Institutional Review Board (IRB# H-47595) and Research and Development Committee of the Michael E. DeBakey VA Medical Center.

Patients included were tested for SARS-CoV-2 infection between 15 February 2020 and 16 June 2021, were hospitalized within 7 days after a SARS-CoV-2 index date, and survived the index hospitalization. The index date was defined as the first SARS-CoV-2 testing date or the hospitalization admission date within 15 days before the SARS-CoV-2 testing date. Hospitalization within seven days after the index date was considered a COVID-19-related admission. Patients with a previous diagnosis of asthma, bronchitis, chronic lung disease, COPD, emphysema, and VTE in the two-year period prior to the index date were excluded [11]. The comparison group, negative (NEG), were patients with negative SARS-CoV-2 test results who were hospitalized within seven days’ index date of testing. We used propensity score matching (PSM) to create the NEG comparison group in the ratio of 1-to-2 (R package MatchIt) [12]. The PSM analysis was adjusted for age (<30, ≥30–<40, ≥40–<50, ≥50–<65, ≥65–<75, ≥75–<85, and ≥85 years), sex (male), and race (White, Black, and Other).

The primary outcomes were a new diagnosis of asthma, bronchitis, chronic lung disease, COPD, emphysema, or VTE within 60 days of index date. The new diagnosis refers to newly documented ICD10 diagnostic codes during inpatient or outpatient encounters post index date of positive test. The new ICD10 codes were not reported in the two years before index date. Mean and standard deviation were calculated for continuous outcomes, and count and percentage were calculated for categorical outcomes. For categorical outcomes, a chi-square test was conducted, and odds ratio (OR) and 95% confidence interval (CI) were computed. Separate logistic regression models were used to calculate the odds of each new respiratory condition with and without adjustments of categorical variables including Charlson Comorbidity Index (CCI ≥ 2), body mass index (BMI ≥ 30), lower respiratory infection history, pneumonia history, ARDS history, and smoking status. The statistical significance was set at 2-sided *p* < 0.05. The statistical analyses were performed using IBM SPSS Statistics version 24 (IBM, Armonk, NY, USA).

## 3. Results

Overall, 1,417,979 patients tested for COVID-19, and 373,048 patents were hospitalized. In total, 161,392 patients met the inclusion exclusion criteria (POS, 18,686; NEG, 142,706). We matched 18,686 POS group with 37,372 NEG group. Participant characteristics are presented in Table 1, including age, sex, race, BMI, and CCI. Compared to the NEG group, the POS group had significantly more cases of asthma (NEG: 402 (1.1%) vs. POS: 294 (1.6%)), bronchitis (NEG: 217 (0.6%) vs. POS: 314 (1.7%)), chronic lung disease (NEG: 4681 (12.5%) vs. POS: 4342 (23.2%)), COPD (NEG: 2502 (6.7%) vs. POS: 1760 (9.4%)), emphysema (NEG: 362 (1.0%) vs. POS: 239 (1.3%)), and VTE (NEG: 1102 (2.9%) vs. POS: 1058 (5.7%)) (Table 2).

The risks of being diagnosed with each respiratory condition were higher in the POS group for asthma (aOR = 1.37 (95% CI = 1.18–1.60)), bronchitis (aOR = 2.81 (95% CI = 2.35–3.36)), chronic lung disease (aOR = 2.14 (95% CI = 2.04–2.24)), COPD (aOR = 1.56 (95% CI = 1.46–1.66)), emphysema (aOR = 1.52 (95% CI = 1.28–1.80)), and VTE (aOR = 1.92 (95% CI = 1.75–2.09)) than in the NEG group (Table 2). The same significant trends were observed in unadjusted ORs. Furthermore, Supplementary Table S1 reports no difference in the risk of respiratory conditions by age and race. Supplementary Table S2 shows ICD10 diagnostic codes.

## 4. Discussion

We analyzed a large national database of veterans hospitalized in VHA with COVID-19 infection and compared them to a propensity score-matched hospitalized cohort with no COVID-19 infection. The data showed that VHA patients hospitalized with COVID-19 had a higher risk of being diagnosed with all respiratory conditions examined, including asthma, bronchitis, chronic lung disease, COPD, emphysema, or VTE compared to hospitalized VHA patients with negative SARS-CoV-2 testing.

Previous observational and case studies have reported the incident rate of developing respiratory conditions in COVID-19 patients hospitalized or discharged. Rashidi et al. have reported a low cumulative rate (0.2%) of symptomatic VTE among 1529 COVID-19 patients within 45 days of hospital discharge [13]. Salisbury et al. have reported that 5.9% of 303 COVID-19 patients were diagnosed with VTE during index hospitalization, and 7.2% were diagnosed with VTE within 90 days of admission [14]. Our results showed that 5.7% of hospitalized COVID-19 patients (n = 18,686) had symptomatic VTE in 60 days of a positive SARS-CoV 19 test. Maestre-Muniz et al., considering 543 patients with COVID-19 at one year after hospital discharge, have reported that 0.4% were diagnosed with asthma, and 1.8% were diagnosed with COPD [15]. Our results showed that 1.6% of veterans hospitalized with COVID had asthma, and 9.4% had COPD within 60 days. Compared to the previous observational and case studies, to the best of our knowledge, this study is the first to explore the independent association between COVID and the development of new respiratory conditions (asthma, bronchitis, chronic lung disease, COPD, emphysema, and VTE) in hospitalized patients with COVID-19 infection. Additionally, the comparison to a matched cohort of patients without COVID-19 infection hospitalized after SARS-CoV-2 testing is a strength of this analysis.

This study has several limitations. First, the cohort is overwhelmingly male and only considers veterans who utilize the VHA for care. Nonetheless, the rich data available from electronic medical record, national distribution of patients, and racial and ethnic diversity are positive attributes of this setting. The diagnosis of respiratory conditions, both for exclusion of those with prior diagnosis and as an outcome, relied on ICD10 codes, introducing the possibility of incomplete or inaccurate classification, as well as the potential for biased documentation. We were unable to review physician notes and other medical record documentation to corroborate the ICD codes or capture additional details about diagnostic certainty, severity, onset, duration, or persistence of these conditions. Future work could address these considerations. Additionally, there is a possibility of missing or under documenting ICD10 codes in the VA EMR. Furthermore, this study is limited by a potential bias in the patient selection process because we could not analyze common diagnoses causing hospitalization. Another study is warranted to consider these limitations.

## 5. Conclusions

Among veterans hospitalized with COVID 19, it is clear that there is an elevated risk of respiratory conditions in the first 60 days after testing. The clinical care team should be alert to this risk and address these conditions in the post-hospitalization management of the patient to reduce the risk of complications and optimize recovery. More targeted clinical research is needed to identify modifiable factors to improve respiratory outcomes after hospitalization for COVID 19.

## Figures and Tables

**Table 1:** Demographics and clinical data for hospitalized veterans with COVID (POS) and matched hospitalized veterans without COVID (NEG).

	NEG	POS
N	37,372	18,686
Age: M; (SD)	66.0 (14.5)	65.8 (14.7)
Age 18–30, N (%)	458 (1.2)	229 (1.2)
Age 30–40, N (%)	2028 (5.4)	1014 (5.4)
Age 40–50, N (%)	2782 (7.4)	1391 (7.4)
Age 50–65, N (%)	10,134 (27.1)	5067 (27.1)
Age 65–75, N (%)	12,134 (32.5)	6067 (32.5)
Age 75–85, N (%)	6338 (17.0)	3169 (17.0)
Age ≥ 85, N (%)	3498 (9.4)	1749 (9.4)
Sex, Male, N (%)	35,136 (94.0)	17,568 (94.0)
Race, N (%)		
White	22,146 (59.3)	11,073 (59.3)
Black	11,568 (31.0)	5784 (31.0)
Other	3658 (9.8)	1829 (9.8)
BMI, Kg/m^2^, M (SD)	29.1 (6.8)	30.1 (6.8)
CCI ≥ 2, N (%)	20,438 (54.7)	10,183 (54.5)

NEG was propensity score matched (PSM) on age, sex, and race (ratio = 1:2). CCI: Charlson Comorbidity Index; BMI: Body mass index; M: mean; SD: standard deviation.

**Table 2:** Comparing potential for new cases of six respiratory conditions by group.

			Odds Ratio (95% Confidence Interval)		
	Negative, N (%)	Positive, N (%)	Unadjusted	Adjusted ^†^	Adjusted ^‡^
Asthma	402 (1.1)	294 (1.6)	1.47 (1.26, 1.71)	1.36 (1.17, 1.58)	1.37 (1.18, 1.60)
Bronchitis	217 (0.6)	314 (1.7)	2.93 (2.46, 3.48)	2.85 (2.39, 3.40)	2.81 (2.35, 3.36)
Chronic lung disease	4681 (12.5)	4342 (23.2)	2.11 (2.02, 2.21)	2.16 (2.06, 2.26)	2.14 (2.04, 2.24)
Chronic obstructive pulmonary disease	2502 (6.7)	1760 (9.4)	1.45 (1.36, 1.54)	1.58 (1.49, 1.69)	1.56 (1.46, 1.66)
Emphysema	362 (1.0)	239 (1.3)	1.33 (1.12, 1.56)	1.54 (1.31, 1.83)	1.52 (1.28, 1.80)
Venous thromboembolism	1102 (2.9)	1058 (5.7)	1.98 (1.81, 2.15)	1.92 (1.76, 2.10)	1.92 (1.75, 2.09)

NEG = negative cases of COVID-19, POS = positive cases of COVID-19. ^†^ Results were adjusted by Charlson Comorbidity index (CCI ≥ 2), body mass index (BMI ≥ 30), and smoking status. ^‡^ Results were adjusted by CCI (≥2), BMI (≥30), smoking status, lower respiratory infection history, pneumonia history, and acute respiratory distress syndrome history.

## Data Availability

The data are available behind the VHA firewall, and they cannot leave the VHA electronic health records. Any request for data access requires official approval process.

## Supplementary Materials

The following supporting information can be downloaded at: https://www.mdpi.com/article/10.3390/healthcare10020300/s1, Supplementary Table S1: Comparing potential for new cases of six respiratory conditions by different age and race. Supplementary Table S2: ICD-10 codes of the respiratory conditions.
