# Supplementary material for: Elevated Risk of Chronic Respiratory Conditions within 60 Days of COVID-19 Hospitalization in Veterans
Source: Healthcare (Basel). 2022 Feb 4;10(2):300. doi: 10.3390/healthcare10020300 (PMC8872176; doi:10.3390/healthcare10020300)
Supplement: Supplementary file 1 [file healthcare-10-00300-s001.zip › healthcare-1564813-supplementary.pdf]

Supplementary Table S1: Comparing potential for new cases of six respiratory conditions by different age and race.

	Asthma	Bronchitis	Chronic lung disease	COPD	Emphysema	VTE
all-ages						
Negative, N (%)	402 (1.1)	217 (0.6)	4,681 (12.5)	2,502 (6.7)	362 (1.0)	1,102 (2.9)
Positive, N (%)	294 (1.6)	314 (1.7)	4,342 (23.2)	1,760 (9.4)	239 (1.3)	1,058 (5.7)
OR(95%CI)	1.47 (1.26, 1.71)	2.93 (2.46, 3.48)	2.11 (2.02, 2.21)	1.45 (1.36, 1.54)	1.33 (1.12, 1.56)	1.98 (1.81, 2.15)
age 18-30						
Negative, N (%)	5 (1.1)	1 (0.2)	27 (5.9)	6 (1.3)	2 (0.4)	8 (1.7)
Positive, N (%)	7 (3.1)	4 (1.7)	36 (15.7)	7 (3.1)	0 (0.0)	11 (4.8)
OR(95%CI)	2.86 (0.90, 9.10)	8.12 (0.90, 73.11)	2.98 (1.76, 5.04)	2.38 (0.79, 7.15)	-	2.84 (1.13, 7.16)
age 30-40						
Negative, N (%)	32 (1.6)	16 (0.8)	134 (6.6)	41 (2.0)	5 (0.2)	51 (2.5)
Positive, N (%)	36 (3.6)	24 (2.4)	200 (19.7)	29 (2.9)	8 (0.8)	40 (3.9)
OR(95%CI)	2.30 (1.42, 3.72)	3.05 (1.61, 5.77)	3.47 (2.75, 4.39)	1.43 (0.88, 2.31)	3.22 (1.05, 9.86)	1.59 (1.05, 2.43)
age 40-50						
Negative, N (%)	57 (2.0)	14 (0.5)	264 (9.5)	78 (2.8)	12 (0.4)	12 (0.4)
Positive, N (%)	41 (2.9)	34 (2.4)	328 (23.6)	84 (6.0)	12 (0.9)	12 (0.9)
OR(95%CI)	1.45 (0.97, 2.18)	4.95 (2.65, 9.26)	2.94 (2.47, 3.51)	2.23 (1.63, 3.05)	2.01 (0.90, 4.48)	2.01 (0.90, 4.48)
age 50-65						
Negative, N (%)	129 (1.3)	57 (0.6)	1,200 (11.8)	596 (5.9)	92 (0.9)	313 (3.1)
Positive, N (%)	108 (2.1)	97 (1.9)	1,210 (23.9)	450 (8.9)	59 (1.2)	289 (5.7)
OR(95%CI)	1.69 (1.31, 2.19)	3.45 (2.48, 4.79)	2.34 (2.14, 2.55)	1.56 (1.37, 1.77)	1.29 (0.93, 1.79)	1.90 (1.61, 2.24)
age 65-75						
Negative, N (%)	102 (0.8)	72 (0.6)	1,702 (14.0)	979 (8.1)	142 (1.2)	346 (2.9)
Positive, N (%)	50 (0.8)	79 (1.3)	1,425 (23.5)	628 (10.4)	93 (1.5)	379 (6.2)
OR(95%CI)	0.98 (0.70, 1.38)	2.21 (1.60, 3.05)	1.88 (1.74, 2.04)	1.32 (1.18, 1.46)	1.32 (1.01, 1.71)	2.27 (1.96, 2.64)
age 75-85						
Negative, N (%)	51 (0.8)	36 (0.6)	869 (13.7)	511 (8.1)	79 (1.2)	183 (2.9)
Positive, N (%)	38 (1.2)	43 (1.4)	756 (23.9)	378 (11.9)	45 (1.4)	184 (5.8)
OR(95%CI)	1.50 (0.98, 2.28)	2.41 (1.54, 3.76)	1.97 (1.77, 2.20)	1.54 (1.34, 1.78)	1.14 (0.79, 1.65)	2.07 (1.68, 2.56)
age >85						
Negative, N (%)	26 (0.7)	21 (0.6)	485 (13.9)	291 (8.3)	30 (0.9)	119 (3.4)
Positive, N (%)	14 (0.8)	33 (1.9)	387 (22.1)	184 (10.5)	22 (1.3)	83 (4.7)

OR(95%CI)	1.08 (0.56, 2.07)	3.18 (1.84, 5.52)	1.77 (1.52, 2.05)	1.30 (1.07, 1.57)	1.47 (0.85, 2.56)	1.42 (1.06, 1.88)
Race-white						
Negative, N (%)	211 (1.0)	153 (0.7)	2,868 (13.0)	1,597 (7.2)	215 (1.0)	568 (2.6)
Positive, N (%)	152 (1.4)	189 (1.7)	2,513 (22.7)	1,030 (9.3)	150 (1.4)	570 (5.1)
OR(95%CI)	1.45 (1.17, 1.79)	2.50 (2.01, 3.09)	1.97 (1.86, 2.09)	1.32 (1.22, 1.43)	1.40 (1.14, 1.73)	2.06 (1.83, 2.32)
Race-Black						
Negative, N (%)	138 (1.2)	46 (0.4)	1,369 (11.8)	686 (5.9)	114 (1.0)	427 (3.7)
Positive, N (%)	108 (1.9)	92 (1.6)	1,395 (24.1)	573 (9.9)	61 (1.1)	379 (6.6)
OR(95%CI)	1.58 (1.22, 2.03)	4.05 (2.84, 5.78)	2.37 (2.18, 2.57)	1.74 (1.55, 1.96)	1.07 (0.78, 1.46)	1.83 (1.59, 2.11)
Race-Others						
Negative, N (%)	53 (1.4)	18 (0.5)	444 (12.1)	219 (6.0)	33 (0.9)	107 (2.9)
Positive, N (%)	34 (1.9)	33 (1.8)	434 (23.7)	157 (8.6)	28 (1.5)	109 (6.0)
OR(95%CI)	1.29 (0.83, 1.99)	3.72 (2.09, 6.62)	2.25 (1.95, 2.61)	1.48 (1.19, 1.83)	1.71 (1.03, 2.84)	2.10 (1.60, 2.76)

NEG = negative cases of COVID-19, POS = positive cases of COVID-19.

Supplementary Table S2: ICD-10 codes of the respiratory conditions.

s	Variable	ICD-10 Code
1	Asthma	J45.20-J45.22, J45.30-J45.32, J45.40-J45.42, J45.50-J45.52, J45.901-J45.902, J45.909, J45.990-J45.991, J45.998, J82, J82.83
2	Bronchitis	J20.0-J20.9, J40, J41.0-J41.1, J41.8, J42, J68.0
3	Chronic lung disease	B44.81, J41.0-J41.1, J41.8, J42, J43.1-J43.2, J43.8-J43.9, J44.0-J44.1, J44.9, J45.20-J45.22, J45.30-J45.32, J45.40-J45.42, J45.50-J45.52, J45.901-J45.902, J45.909, J45.990-J45.991, J45.998, J68.4, J70.1, J81.1, J82, J84.03, J84.112, J98.3, M05.10, M05.111-M05.112, M05.119, M05.121-M05.122, M05.129, M05.131-M05.132, M05.139, M05.141-M05.142, M05.149, M05.151-M05.152, M05.159, M05.161-M05.162, M05.169, M05.171-M05.172, M05.179, M05.19, M30.1, Q32.2-Q32.4, Q33.0-Q33.6, Q33.8-Q33.9
4	COPD	J40, J41.0-J41.1, J41.8, J42, J43.0-J43.2, J43.8-J43.9, J44.0-J44.1, J44.9, J47.0-J47.1, J47.9
5	Emphysema	J43.0-J43.2, J43.8-J43.9, J98.2-J98.3, P25.0, P25.8, T79.7XXA, T79.7XXD, T79.7XXS, T81.82XA, T81.82XD, T81.82XS
6	VTE	I26.01-I26.02, I26.09, I26.90, I26.92-I26.94, I26.99, I80.10-I80.13, I80.201-I80.203, I80.209, I80.211-I80.213, I80.219, I80.221-I80.223, I80.229, I80.231-I80.233, I80.239, I80.241-I80.243, I80.249, I80.251-I80.253, I80.259, I80.291-I80.293, I80.299, I82.210, I82.220, I82.290, I82.401-I82.403, I82.409, I82.411-I82.413, I82.419, I82.421-I82.423, I82.429, I82.431-I82.433, I82.439, I82.441-I82.443, I82.449, I82.451-I82.453, I82.459, I82.461-I82.463, I82.469, I82.491-I82.493, I82.499, I82.4Y1-I82.4Y3, I82.4Y9, I82.4Z1-I82.4Z3, I82.4Z9, I82.621-I82.623, I82.629, I82.A11-I82.A13, I82.A19, I82.B11-I82.B13, I82.B19, I82.C11-I82.C13, I82.C19
